# Editorial: Engineered Nanoporous Materials for Chemical Sensors and Biosensors

**DOI:** 10.3389/fchem.2020.595931

**Published:** 2020-11-12

**Authors:** Abel Santos, Lluis F. Marsal, Tushar Kumeria

**Affiliations:** ^1^School of Chemical Engineering and Advanced Materials, The University of Adelaide, Adelaide, SA, Australia; ^2^Institute for Photonics and Advanced Sensing, The University of Adelaide, Adelaide, SA, Australia; ^3^Australian Research Council (ARC) Center of Excellence for Nanoscale BioPhotonics, The University of Adelaide, Adelaide, SA, Australia; ^4^Department of Electronic, Electric, and Automatics Engineering, Rovira i Virgili University, Tarragona, Spain; ^5^School of Materials Science and Engineering, University of New South Wales, Sydney, NSW, Australia

**Keywords:** nanoporous materials, structural engineering, chemical sensing, biosensing, nanoscience

The current coronavirus crisis has painfully and starkly brought to the surface the intrinsic limitations of benchmark analytical techniques for large-scale, comprehensive screening of populations worldwide. The two main types of SARS–CoV−2 tests available are: (i) RNA-based detection tests by reverse transcriptase polymerase chain reaction (PCR); and (ii) serology tests for IgM and/or IgG antibodies. The former sensing technique provides a direct means of detecting viruses in biological fluids, while the latter test diagnoses the disease indirectly by quantifying the response of the immune system upon viral infection. However, both benchmark techniques have technical constraints. PCR requires highly trained personnel to be operated, large capital investment (>$0.5–1.0 M), has time-consuming sample preparation processes (~24 h per analysis), and high running costs (~$200 per analysis). In contrast, point-of-care serology tests are cost-competitive (~$15–30 per test), provide rapid results with a short sample preparation process (~15–30 min positive/negative), and are easy to use—similar to common pregnancy tests. Despite these advantages, the window period between virus infection and the production of IgM and IgG antibodies (~2 weeks) severely constrains the sensitivity and specificity of serology tests for early diagnosis of SARS–CoV−2 infection. SARS–CoV−2 is an emerging, fast-developing viral disease. So the reliability of SARS–CoV−2 tests is uncertain due to the limited availability of comprehensive databases. Currently performance evidence relies mainly on symptomatic patients, but efficacy in detecting asymptomatic carriers (~40% of cases) remains unclear. We urgently need novel sensing technologies that provide highly selective, high-throughput, on-site, cost-effective, reliable, rapid detection, and molecular fingerprinting of biomolecules to address concerning health threats to our society and their concomitant impact on our economy. Advances in nanotechnology are enabling sensing systems capable of analyzing specific chemical and biological analytes for diagnosis applications, harnessing distinct transduction approaches.

In this context, this Research Topic collates a series of illustrative examples on several chemical sensing and biosensing applications of nanoporous materials. Balderas-Valadez et al. demonstrated a “one spot–two sensors” approach using gold-coated porous silicon interferometers as transduction sensing platforms combining reflectometric interference and surface plasmon resonance. Rajeev et al. engineered a highly sensitive and selective electrochemical biosensor for flightless I protein detection in chronic wound-derived fluids, using chemically-modified porous alumina membranes as electrochemical transductors. Lee et al. presented an interesting review analyzing the application of emerging two-dimensional materials for the selective detection of ions and molecules in liquid samples. Ramos-Ramón et al. developed an interesting sensing system for CO_2_ gas at distinct temperatures by combining ZnO-coated porous silicon optical transducers functionalized with nitrogen-doped carbon dots. Finally, Abu-Thabit and Ratemi presented a mini review compiling recent advances in the development of hybrid plasmonic–fluorescent porous silicon platforms for biosensing applications. These studies are clear examples of technological advances that could provide alternative and complementary analytical tools to overcome the intrinsic constraints of benchmark diagnostic methodologies.

Finally, we would like to thank all authors for their contributions to this Research Topic and all referees for their valuable comments, suggestions, and time, as well as the editorial office for their constant and swift support throughout the editing process.

## Author Contributions

AS, LM, and TK wrote, edited, and revised the manuscript. All authors read and approved the final version of the manuscript.

## Conflict of Interest

The authors declare that the research was conducted in the absence of any commercial or financial relationships that could be construed as a potential conflict of interest.

